# Investigations on the Influence of the In-Stream Pylon and Strut on the Performance of a Scramjet Combustor

**DOI:** 10.1155/2014/309387

**Published:** 2014-08-31

**Authors:** Hao Ouyang, Weidong Liu, Mingbo Sun

**Affiliations:** Science and Technology on Scramjet Laboratory, National University of Defense Technology, Changsha, Hunan 410073, China

## Abstract

The influence of the in-stream pylon and strut on the performance of scramjet combustor was experimentally and numerically investigated. The experiments were conducted with a direct-connect supersonic model combustor equipped with multiple cavities. The entrance parameter of combustor corresponds to scramjet flight Mach number 4.0 with a total temperature of 947 K. The research results show that, compared with the scramjet combustor without pylon and strut, the wall pressure and the thrust of the scramjet increase due to the improvement of mixing and combustion effect due to the pylon and strut. The total pressure loss caused by the strut is considerable whereas pylon influence is slight.

## 1. Introduction

The scramjet generally denotes the ramjet whose flight speed is Ma > 6. The core technology is supersonic combustion, which is a key and difficult issue on developing hypersonic technology. It has been considered for applications on hypersonic cruise missiles, hypersonic planes, aerospace planes, and single stage launchers to orbit. Because of its military and political significance, many countries have made huge investments into scramjet research. The scramjet shows the most potential for air-breathing hypersonic propulsion; however the design of a scramjet combustor is a great challenge. Its performance depends largely on the mixing process, fuel properties, and the supersonic flow throughout the scramjet combustor. A lot of research has already been carried out aiming at achieving efficient mixing between fuel and the core supersonic flow and making optimal conversion of chemical energy into heat. The main studied methods include normal and oblique wall injection [1–5], use of ramp [6–11], pylons [12, 13], aeroramps [14–17], cavities [18–21], swirl [22, 23], struts [24–26], pulsed jets [27, 28], counterflow [29], and shock/shear layer interaction [30–32]. The methods applied in this paper include normal wall injection and the use of struts and pylons. This paper presents the results of experimental and numerical investigations on the influence of in-stream pylon and strut on the performance of scramjet combustor.

## 2. Experimental Apparatus

**Figure 1:**
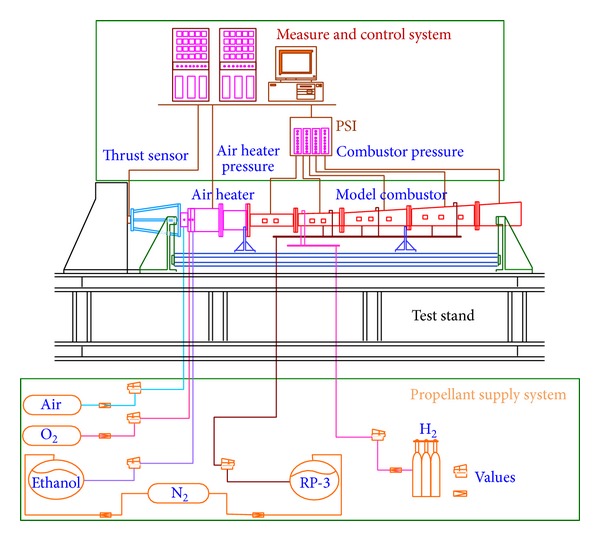
Schematic diagram of the test bench.

The direct connected supersonic combustion ground test system used in this work can be seen in Figure 1. The model scramjet combustor is directly mounted downstream the supersonic nozzle of the air heater which heats the air by means of air/ethanol/O_2_ combustion. The flow conditions of the supersonic nozzle exit, that is the scramjet combustor entry, are listed in Table 1.

**Figure 2:**
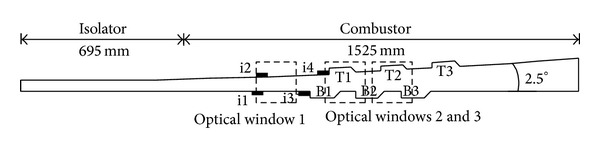
Schematic diagram of the scramjet combustor model.

**Figure 3:**
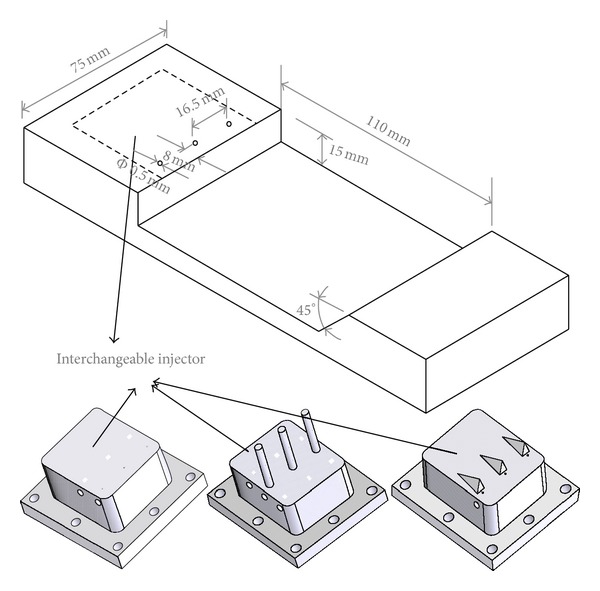
Schematic diagram of interchangeable injectors and a cavity module.

**Figure 4:**
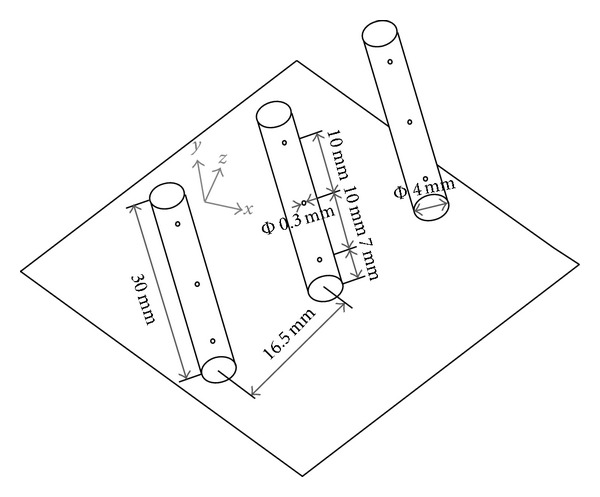
Schematic diagram of struts.

**Figure 5:**
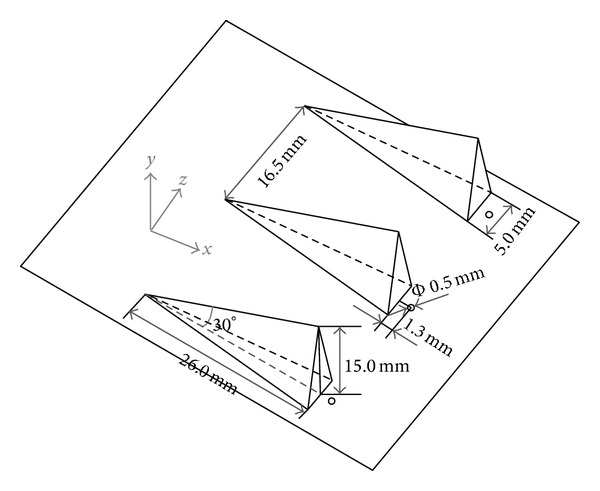
Schematic diagram of pylons.

As Figure 2 shows, the model scramjet combustor consists of a constant cross-section isolator and a one-sided divergent combustor. The cross-sectional area of the isolator is 54.5 mm × 75 mm. The combustor has an expansion angle of 2.5° on the top wall. Six uniform flame-holding cavities are arranged in the divergent section. The cavity size has been given in Figure 3. Here for brevity we denote the interchangeable injector installed position as i1, i2, i3, and i4. The three kinds of interchangeable injector have been shown in Figure 3. In order to avoid the in-stream pylon and strut to burn out, they are installed before the combustor in i1 or i2 positions and will be replaced by the wall normal injector when contrastive experiments without pylons and struts were carried out. In positions i3 and i4, wall normal injectors were applied all the time. Figures 4 and 5 show the schematic diagram and the sizes of the struts and pylons used in this paper, respectively. The kerosene was injected through the holes in the struts and in front of the pylons.

The pressures of combustor along the centerline of the top wall are measured by a series of strain-gauge pressure transducers through taps with the diameter of 0.5 mm distributed on the top wall. The combustion flow field is visualized by high speed imaging camera through three optical windows shown in Figure 2, the flame images were captured through the optical windows 2 and 3, and the schlieren images were captured through the optical window 1. A thrust sensor was used to measure the thrust changes during the experiments.

## 3. Results and Discussion

### 3.1. Results and Discussion about Strut

**Figure 6:**
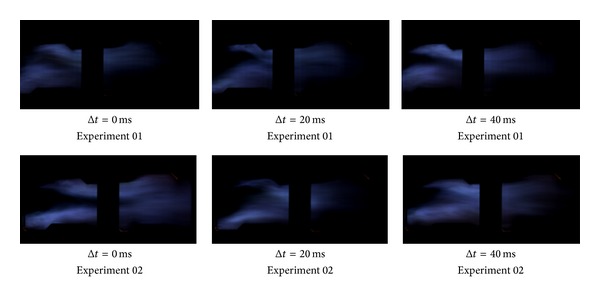
Comparison of high-speed flame images of stable combustion between experiments 01 and 02.

**Figure 7:**
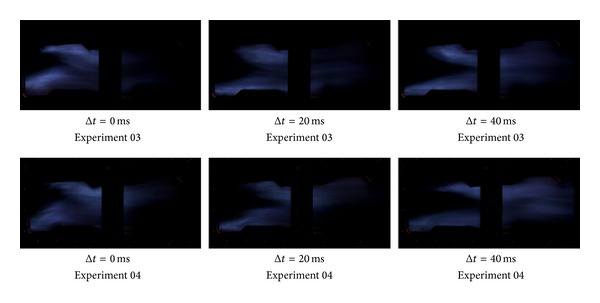
Comparison of high-speed flame images of stable combustion between experiments 03 and 04.

**Figure 8:**
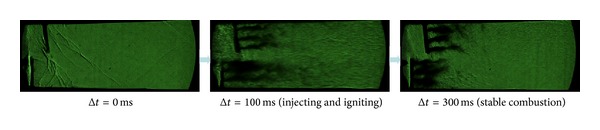
High-speed schlieren images of experiment 05.

**Figure 9:**
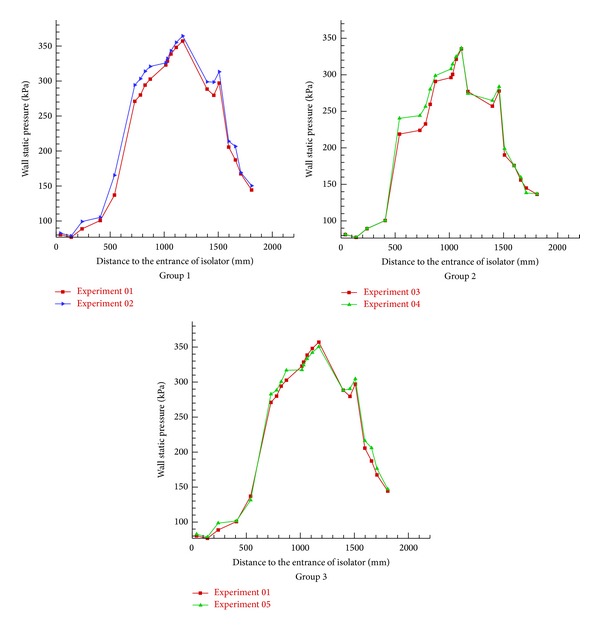
Comparison of wall pressure in strut experiments.

The three group kerosene supersonic combustion comparative experiments between strut and normal wall injection are listed in Table 2. Results showed in Table 2 indicate that a strut can increase the thrust of the model scramjet combustor whether the strut is installed on the top side or on the bottom side or on both sides. As Figures 6, 7, and 9 show, the flame is brighter and the combustion zone is wider; the wall pressure is higher when applying strut, which can indicate that the combustion as well as heat release of kerosene is more adequate.  Figure 8 shows schlieren images of experiment 05. It can be found in it that the heat release of combustion shortens the kerosene atomization and evaporation distance significantly and slows down the core supersonic flow, which has been verified by the disappearance of shock waves. Further studies on the mechanism about enhancing kerosene supersonic combustion by strut have been carried on through three-dimensional numerical simulation. The numerical method has been well described in our former work [33], so it is omitted here. The experiments 01 and 02 are selected for calculation.

Mass-averaged mixing efficiency is defined as follows:

(1) $$\begin{array}{c} \eta_{m}=\frac{{\dot{m}}_{\text{fuel},\text{mixed}}}{{\dot{m}}_{\text{fuel},\text{total}}}=\frac{\int \alpha_{\text{react}}\rho udA}{\int \alpha \rho udA}, \end{array}$$

where

(2) αreact={αα≤αstoic,αstoic1−α1−αstoicα>αstoic,αstoic= m˙fuel,totalm˙air,total + m˙fuel,total,

where *α* is fuel mass fraction. A value of *η*_*m*_ = 0 corresponds to a perfectly segregated state, while *η*_*m*_ = 1 corresponds to a perfectly mixed system.

The total pressure recovery coefficient defined as *ϖ* provides a recovery coefficient of mass-averaged total pressure for a given field and is represented in the equation as follows: $\varpi =\bar{P}/P_{0}$, where $\bar{P}$ is the mass-averaged total pressure for a region of interest and is defined as $\bar{P}=\int \rho uPdA/\int \rho udA$ and *P*_0_ is the freestream total pressure.

**Figure 10:**
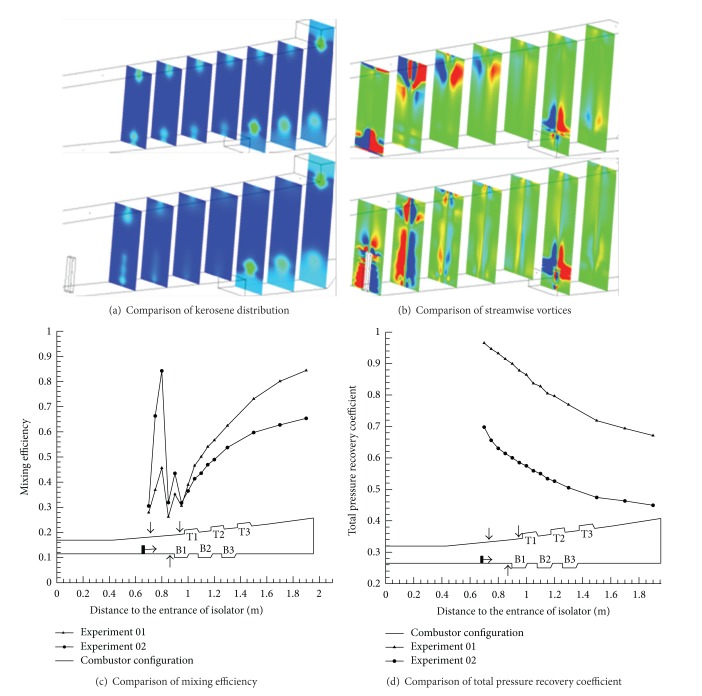
Comparison of calculation results between experiments 01 and 02.

Figure 10 shows the calculated results. Panel (a) shows that struts can result in more uniform distribution of kerosene and panel (b) reveals the disturbance caused by a strut to the core flow will induce additional streamwise vortices. This will improve the mixing effect of fuel and the core flow inevitably, which is verified quantitatively by Figure 10(c).  Figure 10(d) shows strut will cause considerable total pressure loss, which is very likely the reason of group 3 that the thrust only increases 7.1% when struts are installed on both sides.

### 3.2. Results and Discussion about Pylon

**Figure 11:**
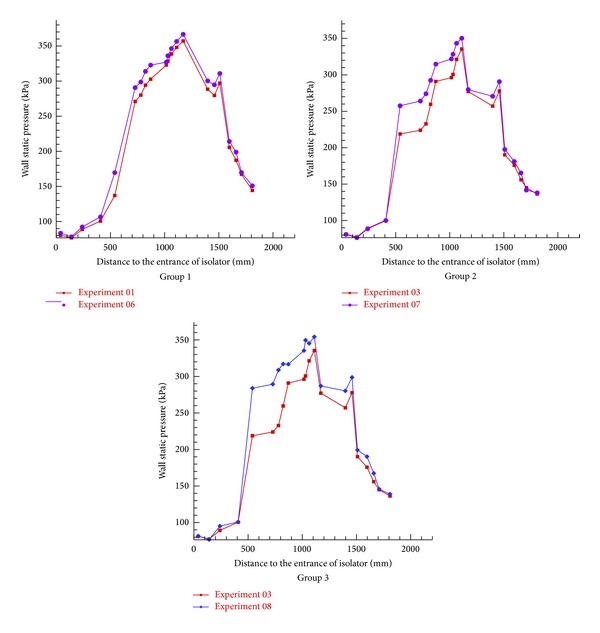
Comparison of wall pressure in pylon experiments.

**Figure 12:**
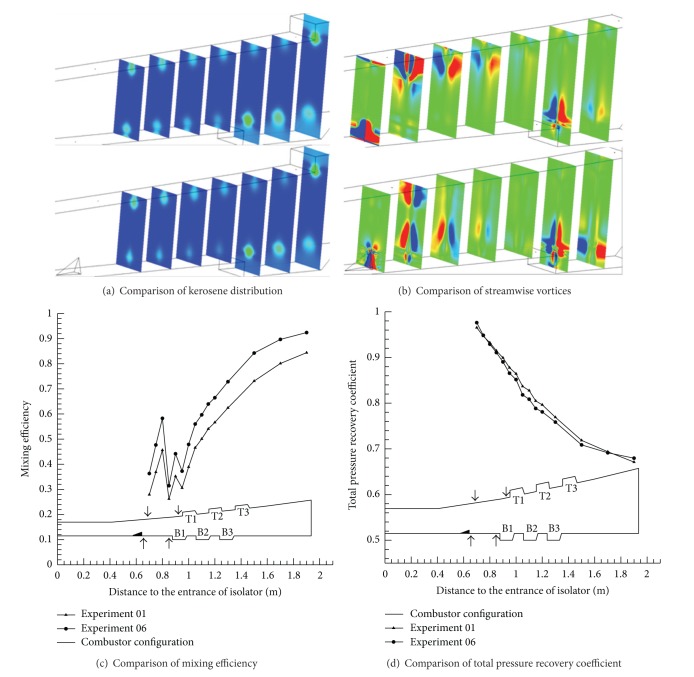
Comparison of calculation results between experiments 01 and 06.

**Figure 13:**
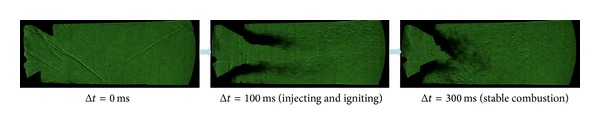
High-speed schlieren images of experiment 08.

The three group kerosene supersonic combustion comparative experiments between pylon and normal wall injection are listed in Table 3. According to Table 3 and Figures 11, 12, and 13, experimental and calculation results about pylons are similar to the ones for struts. In order to avoid repetition, this subsection will put emphasis on the difference between strut and pylon. Firstly, as Figure 12(d) shows, unlike strut, the total pressure loss caused by pylon is slight, so the scramjet thrust will be significantly heightened 20.6% when pylons are mounted on both sides. Secondly, comparison of Tables 2 and 3 shows that the effect of strut and pylon when mounted on top side is similar but strut is better with respect to bottom side. Additionally, according to Figure 13, we can find, unlike the parallel fuel injection of strut, that the penetration quality of the transverse kerosene jets behind the pylon will be improved so much that the two jets will interact, which can boost the break of kerosene droplets to improve the atomization, evaporation, and mixing effect of kerosene.

## 4. Conclusion

In the present study, experiments and three-dimensional numerical simulations were conducted using kerosene as fuel to study the effect of pylon and strut on enhancing mixing and combustion in scramjet flight with Mach number 4.0. Based on the present results, a few conclusions can be drawn.

1. Both strut and pylon can increase the mixing efficiency of fuel and main flow and enhance the kerosene combustion to improve the performance of a scramjet combustor whether mounted on the top side or on the bottom side or on both sides.
2. Strut can optimize the fuel distribution and generate additional streamwise vortices by the disturbance to the main flow.
3. Pylon can also generate streamwise vortices. But unlike the parallel fuel injection of strut, it is probable to lead to transverse jet interaction by improving the penetration quality of jet.
4. Strut results in considerable total pressure loss, whereas the total pressure loss caused by pylon is slight.

## Figures and Tables

**Table 1:** Flow conditions at the scramjet combustor entry.

Ma	*P*/KPa	*T*/K	*P*_0_/MPa	*T*_0_/K	*Y*_O_2__
2.1	71	528	0.65	947	23.3%

**Table 2:** Comparison experiment about strut.

Group number	Exp. number	Position of strut	*ϕ*	Thrust/*N*	Thrust gain
1	01	Without	1.10	1110	11.9%
	02	i1		1242	

2	03	Without	0.88	980	10.7%
	04	i2		1085	

3	01	Without	1.10	1110	7.1%
	05	i1, i2		1189	

**Table 3:** Comparison experiment about pylon.

Group number	Exp. number	Position of pylon	*ϕ*	Thrust/*N*	Thrust gain
1	01	Without	1.10	1110	9.5%
	06	i1		1215	

2	03	Without	0.88	980	10.6%
	07	i2		1084	

3	03	Without	0.88	980	20.6%
	08	i1, i2		1182	
